# Surface enhanced deep Raman detection of cancer tumour through 71 mm of heterogeneous tissue

**DOI:** 10.7150/ntno.71510

**Published:** 2022-03-21

**Authors:** Priyanka Dey, Alexandra Vaideanu, Sara Mosca, Marzieh Salimi, Benjamin Gardner, Francesca Palombo, Ijeoma Uchegbu, Jeremy Baumberg, Andreas Schatzlein, Pavel Matousek, Nick Stone

**Affiliations:** 1 School of Physics and Astronomy, University of Exeter, Exeter EX4 4QL, UK; 2 School of Health and Life Sciences, Teesside University, Middlesbrough TS1 3BX, UK; 3 School of Pharmacy, University College London, London, UK; 4 Central Laser Facility, Research Complex at Harwell, STFC Rutherford Appleton Laboratory, UK Research and Innovation, Harwell Campus OX11 0QX, UK; 5 NanoPhotonics Centre, Cavendish Laboratory, Cambridge, CB3 0HE, UK

**Keywords:** Plasmonic gold nanoparticles, SERS, SORS, TRS, SESORS, SEDRS mouse breast cancer, cancer tumour detection.

## Abstract

Detection of solid tumours through tissue- from depths relevant to humans- has been a significant challenge for biomedical Raman spectroscopy. The combined use of surface enhanced Raman scattering (SERS) imaging agents with deep Raman spectroscopy (DRS), i.e., surface enhanced deep Raman spectroscopy (SEDRS), offer prospects for overcoming such obstacles. In this study, we investigated the maximum detection depth through which the retrieval of SERS signal of a passively targeted biphenyl-4-thiol tagged gold nanoparticle (NP) imaging agent, injected subcutaneously into a mouse bearing breast cancer tumour, was possible. A compact 830 nm set-up with a hand-held probe and the flexibility of switching between offset, transmission and conventional Raman modalities was developed for this study. *In vivo* injection of the above SERS NP primary dose allowed surface tumour detection, whereas additional post mortem NP booster dose was required for detection of deeply seated tumours through heterogeneous animal tissue (comprising of proteins, fat, bone, organs, blood, and skin). The highest detection depth of 71 mm was probed using transmission, translating into a ~40% increase in detection depth compared to earlier reports. Such improvements in detection depth along with the inherent Raman chemical sensitivity brings SEDRS one step closer to future clinical cancer imaging technology.

## 1. Introduction

Recent developments in Raman spectroscopy have opened new potential directions for *in vivo* non-surgical cancer diagnosis.^1^, ^2^ For example, inherent cancer biomarkers such as microcalcifications, especially in the breast, have been successfully detected by deep Raman spectroscopy (DRS) in transmission modality (TRS) through ~40 mm tissue thickness.^3^ This was feasible because the microcalcifications exhibit distinct Raman signals differing from those of the soft tissues found in the breast. Nevertheless, microcalcifications may not always be present at every cancer site, thereby making it essential to develop and utilize alternative Raman reporters to differentiate the cancer site from the surrounding soft tissues. Plasmonic gold nanoparticles (NPs) act as carriers of the Raman label and cancer targeting ligand, ensuring cancer site detection via the reporter. Furthermore, gold NPs behave as Raman signal amplifiers for the reporter molecules (tag or label) positioned close to the gold nanosurface, a phenomenon popularly known as surface enhanced Raman scattering (SERS).^4^-^6^ Such SERS-labelled gold nanostructures can act as contrast (or imaging) agents by emitting signals from the co-located tumour. This is possible as these agents, when injected into an organism, can actively or passively target the cancer site and preferentially accumulate at the cancer site. Tracking the spectral signatures of the SERS label thereafter allows the determination of the absence/presence and location of the tumour.^7^ By combining SERS-labelled gold nanostructures and deep Raman spectroscopy, surface enhanced deep Raman spectroscopy (SEDRS) has allowed increasing the depth detection limits of tumour in tissue.^8^-^11^

The concept of DRS was first demonstrated by Matousek and co-workers;^12^ where they implemented a physical offset of a few millimetres between the laser excitation and the backscattered signal collection (often denoted as 'Δs'), introducing spatially offset Raman spectroscopy (SORS), to detect sub-surface signals. Different set-ups or modalities have been explored since then under the umbrella of DRS, to probe deeper and enhance the signal ratio of sub-surface to the top surface composition. Some strategies have also been deployed to introduce flexibility in the spatial offset instrumentation. They include the ability to move the backscattered point-like collection (SORS) from the point-like excitation for variable offset; introducing an axicon lens in the illumination pathway, thereby converting the point-like excitation into a ring shape with backscattered collection positioned at the centre of the ring (inverse SORS, Δs = radius of the ring); or using both point-like excitation and collection in a transmission modality (TRS).^13^ Although different modalities have been proposed only a few reports have attempted their comparison. For example, Conti *et. al.*^14^ compared different modalities of micro-SORS and Mustafa *et. al.*^15^ has studied the difference between diffuse and inverse SORS. Therefore, evaluating different modalities for deep Raman diagnosis would be highly beneficial to enable end-user specific SEDRS modality selection. Reports of investigating the highest achievable depth of a model tissue system have also been explored.^16^, ^17^ To this end, various SERS nanostructures have been employed to boost the SERS signal, including shaped nanoparticles (NPs)^7^, nano-assemblies^6^, ^10^, ^18^ or NIR-resonant SERS labels^9^, a detailed account can be found in a recent review by Dey and co-workers.^19^ The SORS and SERS techniques have first been combined and described as SESORS in a study by Stone *et al.*^8^ and further expanded to demonstrate multiplexed SESORS imaging through 50 mm porcine tissue in transmission SEDRS modality.^20^

Critical studies that employ the above approaches for SEDRS detection from tissue depths have been elaborated below. Sharma and co-workers^21^ have demonstrated the ability to distinguish between different neurotransmitters functionalized onto gold nanostructures through 2 mm of cat skull. This SESORS detection has been achieved when employing a 785 nm laser, and 2 mm SORS offset (i.e., point-illumination and ring collection). Dey *et al.* used a similar 2-3 mm SORS offset (point excitation and ring collection) and 785 nm laser illumination^10^ have reported SESORS detection from a depth of 8 mm from within protein-rich animal tissues. This study, for the first time, employed sub-100 nm gold nano-assemblies (NPs glued together with polymer linkers into a colloidally stable morphology) and experimentally demonstrated the benefit of using nano-assemblies to that of single gold NPs, both labelled with 2-quinolinethiol SERS label. In addition to nanostructure design and SORS offset set-ups, researchers have explored the potential of enhancing the SERS signals further by utilizing molecules including trans-1,2-bis(4-pyridyl)-ethylene (BPE) and near-infrared NIR resonant dyes. For example, van Duyne and co-workers^21^ have shown that a nanostructure concentration of 10^12^-10^13^ NPs decorated with a strong SERS label (e.g. BPE) could allow detection through 8 mm of porcine bone, using a 785 nm laser with 2-3 mm backscattered SORS offset. Faulds and co-workers^9^, ^22^ have systematically shown the design and application of NIR-absorbing SERS labels for resonant SESORS with 830 nm laser excitation. They have reported PCA-distinguishable detection from a significant depth of 48 mm^19^ of porcine tissue when using an 8 mm offset with a commercial SORS set-up. Importantly, they have shown that a resonant NIR label 'dye 823' provides about 40 times stronger signal than BPE.^9^ To date, the highest depth achieved with SEDRS has been reported by Stone *et al.*^23^ where non-resonant Oxonica/Cabot NPs (100 nm gold spherical NPs functionalized with SERS label 5-(4-pyridyl)-1,3,4-oxadiazole-2-thiol or POT encapsulated in silica) could be detected from within 50 mm of porcine tissue in TRS modality. Detection was possible using 3×10^10^ NPs with 300 s accumulation employing an 830 nm laser and 210 mW power at the sample surface. All the above studies have reported detection from either nanoparticle colloids in cuvettes buried within tissues or injection of the NP colloid directly at different tissue depths. Recently, Nicolson *et al*.^7^ demonstrated *in vivo* Raman SESORS detection of brain tumour through a mouse skull. They employed resonant SERS label 'IR792' functionalized onto gold nanostars and used 785 nm laser excitation and 2.5 mm SORS offset. Nevertheless, achieving an order of magnitude higher depth is likely to be needed for enabling tumour detection in humans. Therefore, gaining access to tissues at greater depths is vital for advancing deep Raman spectroscopy as a clinical cancer diagnosis tool in human subjects.

Researchers in the field of SEDRS are limited by the small size of the majority of animal models available for *in vivo* studies. This results in most deep Raman studies being demonstrated to date in *ex vivo* animal tissues or phantoms to exploit the SEDRS depth detection capabilities fully. We attempted to bridge the *in vivo* and *ex vivo* animal models typically studied and investigated a scenario that demonstrates the SEDRS depth capabilities and serves as an initial demonstrator towards a more human-relevant scenario. In this study, SEDRS depth detection was performed post mortem by wrapping the whole mouse in a 'blanket' of protein-rich porcine tissue to increase the overall detection depth beyond the mouse's natural body thickness of 15 mm, thus enabling us to bridge both *in vivo* whole animal model and thicker *ex vivo* tissue models. The SERS NPs (i.e., biphenyl-4-thiol (BPT)-functionalized, PEGylated spherical gold NPs) were injected in a two-step process with a primary dose of NPs provided as an *in vivo* subcutaneous (sc) injection. This enabled initial surface tumour detection. This was followed by a booster dose of identical NPs by providing a post mortem subcutaneous injection, demonstrating the detection of more deeply seated tumours. This aided the demonstration of maximum depth probed by the SEDRS system for tumours at different tissue depths critically through a heterogeneous animal tissue model comprising of protein, lipids, skin, bone, organs, and blood. Importantly, we achieved tumour detection through a depth of 71 mm in the SEDRS transmission modality, approximately 40% increase in measured depth compared to earlier state-of-the-art report in SEDRS detection.

## 2. Results and Discussion

For Raman spectroscopy to enable diagnosis and monitoring of cancers beneath the surface, development in some key areas is essential, including compact instrumentation and sensitive detection particularly from heterogeneous tissue at human-comparable tissue depths with animal models. Our study aimed at addressing these challenges.

### 2.1 Developing the SEDRS capability i.e, designing DRS instrument and SERS NPs

Firstly, we focused on developing a handheld deep Raman probe with a small footprint, similar to an ultrasound probe, with multiple SEDRS functionalities (point, inverse SORS and transmission modalities) that could be important for diagnosis. Switching between the different available DRS modalities could be done easily, without disturbing the sample. The choice of the laser wavelength is critical. The near infrared (NIR, 750-900 nm) wavelengths provide lower scattering coefficient and higher total attenuation length in the tissue and hence is the typical laser range of choice.^19^ Furthermore, Ghita *et al.*^24^ suggested that a laser of 790-810 nm was an optimum illumination source for detecting microcalcifications with transmission Raman (Raman signal typically, at ~960 cm^-1^) after investigating excitation wavelengths from 770-830 nm. Given the available laser wavelength in this set-up, we settled on an excitation line of 830 nm, reasonably close to the optimum range. The set-up used an 830 nm laser from Innovative Photonic Solutions, USA delivering ~350 mW power at sample surface (see Methods section for details). The configuration for the different modalities could be modified by either changing the position of the collection arm (fibre bundle) or by moving the axicon lens in and out of the light path. The three operating modalities were: (a) conventional backscattered point-modality where the illumination and collection are both point-like and represents the same spot (Δs=0), (b) inverse SORS modality with a ring illumination and point collection (Δs=5 mm) (see Supporting Information, figure S1), and (c) transmission modality (see Supporting Information, figure S2).

To demonstrate the capabilities of the in-house built system for SEDRS detection, SERS-labelled gold nanoparticles (NPs) were employed. The spherical gold NPs (nanoComposix, USA) of 60 nm diameter, featuring a localized surface plasmon resonance peak at 534 nm, were labelled with biphenyl-4-thiol (BPT). The SERS nanostructure thus prepared was non-resonant to the near-infrared 830 nm laser employed for the study. The nanostructure was further enveloped in a linear polyethylene glycol (PEG) polymer coating to impart 'stealth' properties when in contact with biological fluids and to minimize clearance by the mononuclear phagocyte system. PEG coating additionally provides steric hindrance to minimize random aggregation of the SERS NPs, necessary for eliminating blood vessel blockage and reducing the variability of the SERS signal intensity. The median hydrodynamic diameter of the BPT-labelled PEGylated gold NPs (see Supporting Information, figure S3) was approximately 90 nm (FWHM of 70-150 nm), allowing it to passively target and accumulate in the cancer tumour.^25^

### 2.2 Surface tumour detection with a primary dose of *in vivo* SERS NP injection

We opted for a subcutaneous (sc) injection route to intravenous (IV) injection^26^, so that the short-range systemic circulation ensured a larger proportion of the injected NP dose to accumulate in the tumour. The passively targeted NPs were injected subcutaneously, *in vivo,* near the 4T1 mammary carcinoma tumour on the right flank of a female BALB/cAnNCrl (see Methods section for details). The mouse weight was about 21 g and the tumour weighed 0.31 g. An initial volume of 200 µL colloidal solution containing a total of 1.5×10^9^ SERS imaging NPs (calculated from UV-visible-NIR spectroscopy, detailed in the Methods section) was used as the primary dose. The mouse was kept alive for 4 hours after injection for short-range systemic circulation, as observed to be optimum for *in vivo* studies of gold nanostructures (unpublished data). The mouse was then euthanized, frozen (UCL, London, UK) and transported to the deep Raman laboratory (UoE, Exeter, UK). A significant fluorescent signal dominated the Raman spectrum from the locations with the hair and therefore removing the hair on and around the tumour was necessary. This was done by carefully using a surgical scalpel on a frozen animal. This was followed by thawing the carcass for 2-3 hours before taking Raman measurements with the different modalities of the in-house built DRS set-up.

**Figure 1:**
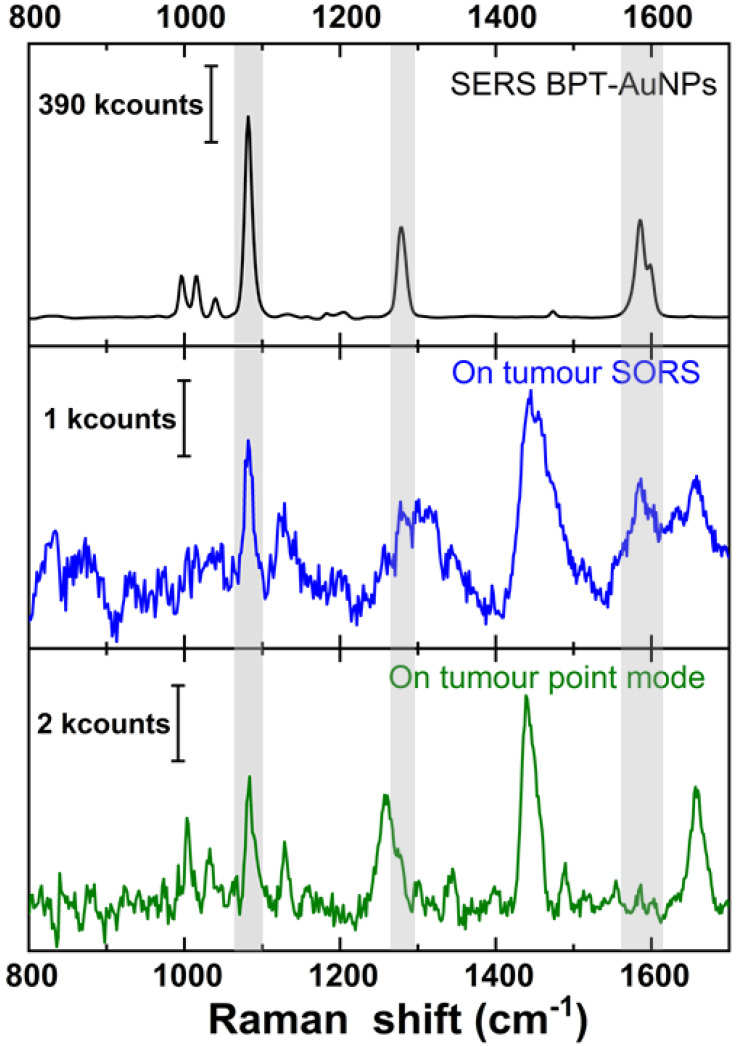
Comparison of (top, black) reference SERS spectrum of gold nanostructure with the signature peak of BPT marked with grey shading, (middle, blue) inverse SORS spectrum (Δs = 5 mm), and (bottom, green) conventional Raman point-spectrum. Middle and bottom spectrum collected with direct laser illumination on mouse tumour, with 60×1 s accumulation, and further background subtracted.

***Tumour detection spatially in the X-Y plane.*** All mouse measurements were performed post mortem on the intact mouse. Initial measurements were carried out by pointing the laser at the naked (hair-free) mouse tumour. Figure 1 (top spectrum) shows a reference SERS spectrum of the BPT-labelled NPs. After subtraction of a polynomial simulated background from the averaged spectrum, the SERS signature peaks of BPT at 1079 cm^-1^ (marked by grey shading) could be identified for both the backscattered point-modality (figure 1 green spectrum) and the inverse SORS modality with a 5 mm offset (figure 1 blue spectrum). This confirms that the BPT-labelled SERS NPs were indeed present at the tumour site. We analyzed the SERS signal from the tissue surface based on the spectra' signal-to-noise (S/N) ratio and the intensity of the 1079 cm^-1^ peak. Additional peaks around 1450 and 1650 cm^-1^ in the observed spectrum are potential contributions from the lipid and protein components of the mouse skin. The inverse SORS provided NP and tumour identification like the conventional Raman point-modality and helped in suppressing signals of the tissue, particularly that of subcutaneous lipid. When comparing SORS and point modality, the inverse SORS presents itself as a better candidate for identifying the presence of the NPs with highly resolved NP peaks while minimizing false positives due to broad tissue signals. A 1.5×10^9^ NP concentration was sufficient to probe detection of the tumour located near the skin surface, relevant in scenarios like human skin cancer or head and neck cancer.

### 2.3 Deeply seated tumour detection with post mortem booster dose of SERS NPs

Precisely locating a deeply seated tumour is more challenging and clinically more important than detecting surface tumours. As the rodent animal model was inherently unable to provide human-relevant tissue depths, combining the animal model with *ex vivo* animal tissue layers was explored. Studies suggest doses of 10^14^-10^15^ NPs of PEGylated gold NPs as a required threshold for saturating the reticuloendothelial system in mouse models and increasing the NP accumulation at the tumour site.^27^ This suggested that the primary SERS NP dose injected *in vivo* of approximately 10^9^ NPs would not be sufficient to probe detection from more significant tissue depths. To this end, a booster dose of the same NP solution, in addition to the primary dose, was thus injected subcutaneously *post mortem* to the same mouse, following the first surface measurements, to investigate the detection of deeply seated tumours. This additional booster dose on the same mouse was done in the spirit of the 3Rs^28^, reducing the need for additional animal sacrifice.

#### 2.3.1 Booster dose post mortem

Based on the following studies, the NP dose that could be used for deeply seated tumours was estimated. In a recent review, the authors determined the median NP tumour accumulation values to be 0.6% for passive targeting, which could increase up to 0.9% for active targeting in mouse models (data from table 1 of Ref. 25 based on data from online repository introduced by Wilhelm *et al.*^29^ containing 238 datasets from 118 publications). Therefore for doses of 10^14^-10^15^ NPs,^27^ this would translate into around 6×10^11^-10^12^ NPs available at the tumour. To achieve a final NP dose similar to this, we used a booster dose (as a top-up to the primary dose) of the identical NP in a concentrated form. A 100 µL containing a total of 7.2×10^11^ NPs (see Methods section for more details) was injected subcutaneously post mortem (near the tumour, into the same mouse used earlier) to yield a combined net injected concentration of less than 10^12^ NPs. Assuming that all NPs were transferred from the syringe to the mouse injection site, a net calculated concentration of 7.22×10^11^ NPs and 160 µg of gold should accumulate in and around the tumour, as no short-range circulation was possible post mortem. Similar concentrations have also been reported for *in vivo* studies of photothermal therapy^30^, ^31^ and in *ex vivo* Raman studies by van Duyne and co-workers^21^. The functionalized gold NP injected sub-cutaneously for the mouse body weight ranged from 155 µg/kg for the primary *in vivo* to about 7400 µg/kg for the booster. Studies with similar gold NP size and dosages to that of the booster dose injected intravenously to male BALB/c mice have been reported to be non-toxic by Chen *et al.*^32^*,* and non-toxic but contributing to decreased collagen synthesis without any impact on cardiac function by Yang *et al*.^33^ In a recent review, Adewale and co-workers have summarized the influence of physicochemical properties and other factors in the toxicological behavior of gold NPs on different *in vitro* and *in vivo* animal models.^34^

**Figure 2:**
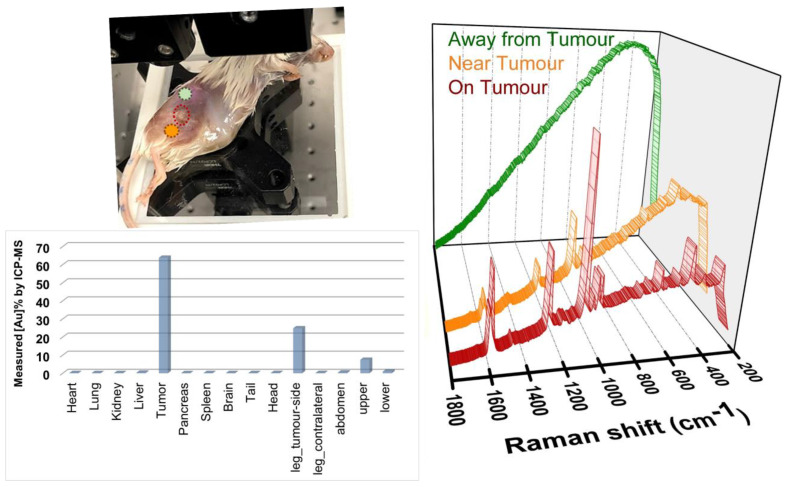
Surface scan spectra acquired on different locations with conventional backscattered point-modality Raman with 1 s acquisition time and 1 accumulation. The green spectrum represents the farthest from the tumour, orange is near the tumour (on the tumour-bearing leg) and the red spectrum represents detection from the tumour. The same Y-axis scale representing intensity in arbitrary counts is used for all spectra.

After all the measurements were completed, the injected animal was dissected and analyzed for the gold concentrations using inductively coupled plasma mass spectroscopy ICP-MS (see details in Methods section, figure 2, and table S1 in Supporting Information). The results show that about 64% of the total gold measured in the mouse was found in the tumour accounting for 7.45×10^9^ NPs, equivalent to a NP density of approximately 52 µg/g in the tumour. Interestingly, the second-highest concentration of NPs i.e., approximately 25% of the total gold, was observed within the tumour-bearing leg or the injection site. In comparison, negligible gold was observed from the contralateral leg (without any tumour). This suggests that some NPs that were injected subcutaneously near the tumour either might not have reached the tumour or have leaked and accumulated in the tumour-bearing leg. Negligible concentrations of gold were detected in the kidney, liver, and spleen which is consistent with short-range systemic circulation supported for subcutaneous injections. Overall, a total gold concentration of 10^10^ NPs and 25.6 µg were found in the mouse. The difference in concentration of total theoretical injection and measured values are combinedly attributed to some NPs not being transferred from the syringe, NPs leaking away through veins or interstitial fluids during freezing and thawing of the mouse (between booster injection and ICP-MS measurements), the effect of NP acid digestion from organs for ICP-MS sample preparations, and the accuracy of ICP-MS measurements. Therefore, the ICP-MS results were used as a guide for evaluating the dose ratios between organs and confirming that the net NP concentration was lower than 10^12^. The net NP doses reported here onwards are based on the calculated NP concentrations as mentioned earlier.

**Figure 3:**
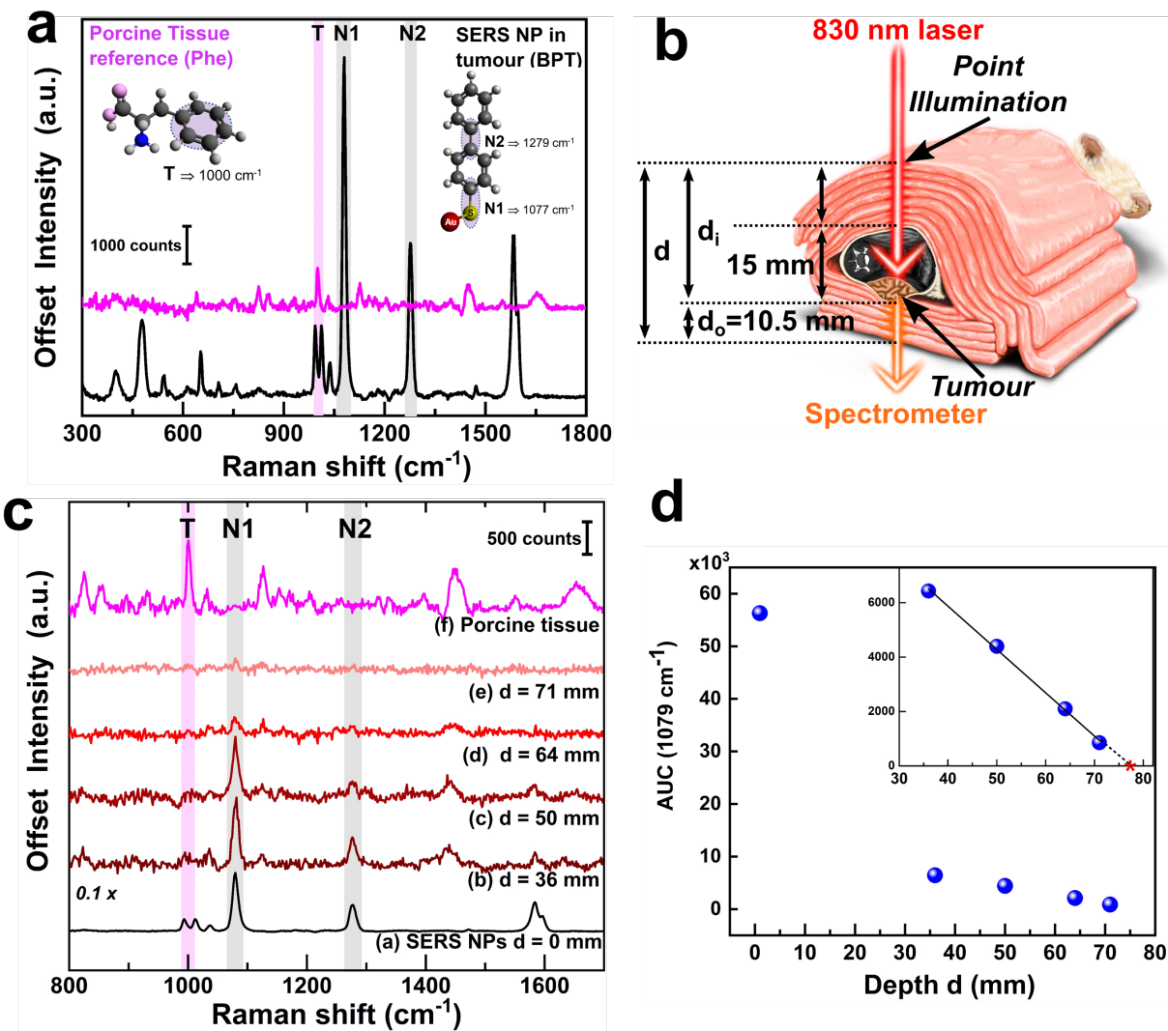
(a) Raman spectrum of porcine tissue (pink) and SERS spectrum of BPT gold NPs (black) along with structural vibrations in the inset, (b) schematic showing the TRS SEDRS measurements, the cross-section shown for better understanding, while all actual measurements were performed on the whole animal, (c) TRS spectra at various depths d with marked peak position from porcine tissue T and BPT-NPs N1 and N2, and (d) area under the curve AUC of peak Y at 1079 cm^-1^ with respect to depth d with zoomed inset of depth 30-80 mm. The linear fit could be expressed as y = 12241-1592x and R^2^ = 0.99758, where substituting y or AUC with a minimum value of 100 for identification, x or depth equals 76.2 mm marked on the inset as with a red star.

The mouse was left undisturbed for 30 minutes after injection before proceeding with the Raman measurements. Surface scan measurements (spectrum measured at multiple locations on the mouse skin) was carried out, as shown in the inset of figure 2 with point modality. This could help corroborate the above ICP-MS data and confirm if a significantly higher proportion of NPs was indeed located at the tumour site with respect to other locations. It was observed that as we moved closer to the tumour (from green spectrum to orange towards red spectrum in figure 2), the background fluorescence decreased while the SERS signal intensity increased. The plot in figure 2 shows that the highest signal intensity of the BPT signature peaks was observed from the tumour (red spectrum), justifying the ICP-MS results of the highest NP concentration at the tumour site. This suggested that a spectral differentiation of the tumour from the surrounding soft tissues was feasible within a scan area of a few mm^2^. As reported by Zhang *et al.*^35^, the BPT signature peaks from the SERS NPs were observed at 1079 cm^-1^ for ν(C-S) (marked as 'N1' theoretical 1077 cm^-1^), as well as at 1276 cm^-1^ that can be assigned to a stretching of the central C-C bond between the phenyl rings (marked as 'N2' theoretical 1279 cm^-1^) as depicted in figure 3a (black spectrum). The BPT signal was also observed in a region very close to the tumour (in the leg - figure 2, orange spectrum) which was probably due to being the site of injection or due to NPs leaking out of the tumour, as discussed earlier. When comparing the positions 'on tumour' and 'near tumour', approximately a 3:1 concentration-dependent signal intensity was observed (figure 2) for a three-times gold concentration as observed by ICP-MS (63.8% for the tumour to that of 24.8% for the leg). No BPT signal was observed even a few mm away from the tumour and leg (figure 2, green spectrum). In contrast, significant fluorescence could be observed due to a combined effect of the absence of strong SERS signal and the presence of tissue fluorescence and some remaining fluorescent white hair on the skin surface. This concentration-dependent signal discrimination within an area of a few mm^2^ would be beneficial for determining the precise tumour position and tumour margin, specifically when attempting multiplexed site detection.

#### 2.3.2 Tumour detection at tissue depth (Z-plane) by transmission DRS

The uncompressed thickness of the mouse was only about 15 mm across its body. Hence, to attempt detection through larger tissue thicknesses, we wrapped the entire post mortem mouse with additional layers of store-bought unprocessed porcine tissue medallions. The reference spectrum of the porcine tissue and that of the BPT-labelled NPs are shown in figure 3a. The porcine tissue (figure 3a, pink spectrum) features a signature peak at 1000 cm^-1^ which can be assigned to the ring breathing mode of phenylalanine^36^ (Phe, denoted with 'T', shown as pink shading). The 1000 cm^-1^ peak is distinguishable from the characteristic doublet observed for BPT at 993 cm^-1^ and 1011 cm^-1^. In contrast, the signature peaks of BPT could be observed at 1079 cm^-1^ (N1) and 1276 cm^-1^ (N2) (figure 3a, black spectrum, grey shading).

For transmission TRS modality measurements, as depicted in figure 3b, the tissue layers were placed such that the laser photons travelled through the thickness 'd_i_' comprising of the porcine tissue layers and mouse body (hair removed, uncompressed thickness of 15 mm including skin, bone, organs, blood) to reach the tumour. The generated Raman photons would then travel through the lower layers of porcine tissue thickness 'd_o_', thus probing detection through a heterogeneous animal tissue of thickness 'd' (where d = d_i_ + d_o_, as shown in figure 3b). The thickness was increased from 36 mm to 71 mm in increments of 3.5 mm. The d_o_ was kept constant at 10.5 mm, while the d_i_ was varied by adding more porcine tissue layers. Three individual measurements with 10 s acquisition time for each depth, with minimal disturbance to the sample, were recorded. Spectra were then averaged (equivalent to a 10 s accumulation). Subsequently, background subtraction was performed (a polynomial background curve fitting in OriginPro, see Methods section for details). Additional raw spectra in figure S5 and spectral post-processing method in figure S6 (in Supporting Information) have been shown for TRS modality. This final unsmoothed spectrum was then analyzed for each depth d. Spectral subtraction of the reference porcine tissue at each depth was avoided as each probed region exhibited varying contributions of BPT Raman signals and tissue fluorescence. This approach allowed us to directly identify the presence/absence of SERS NPs, independent of the varying spectral signature of the heterogeneous tissue, a scenario beneficial for real-world applications. Figure 3c depicts that the SERS intensity of the NPs was inversely related to the thickness d, while the signature peak from the porcine tissue at T remained largely unaltered with significantly lower intensity (figure 3c, spectrum b to e). Detection could thus be experimentally probed using TRS from a significant depth d of 71 mm with a signal-to-noise ratio SNR of 2.11 (spectrum e). Further analysis was carried out using the area under the curve (AUC) for peak N1 at 1079 cm^-1^ which was calculated by summing the intensity over the polynomial background curve. This was plotted against depth d in figure 3d, which suggested a dramatic drop in AUC values from the point-modality (i.e., d=0 mm) to TRS modality (d=36 mm) (figure 3d) initially, followed by a linear decrease of AUC with the total TRS depth d (figure 3d inset). The linear fit was expressed by y = 12241-1592x (y=AUC and x=depth d) with R^2^ of 0.99758. Considering a theoretical minimum SNR of 2 with an assumption of a minimum AUC of 100 necessary for positive identification of the SERS NPs, the above equation results in the theoretical limit of detection depth d of 76.2 mm.

The experimental detection thickness achieved in this study 71 mm is about 40% higher, and the theoretical limit of detection of 76 mm is about 50% higher than that reported earlier which was 47-50 mm by commercial SERS Oxonica NPs with TRS modality by Stone* et al.*^23^. Therefore, this study reports the highest detection depth in an animal model to date with any SEDRS system. This significant improvement can be attributed to a combination of factors, including higher SERS NP density within a 3D tumour volume reducing the scope of NPs leaching out from the detection volume, improved SEDRS optics with relatively higher laser power and improved signal collection.

The observed detection depth of 71-76 mm is comparable to porcine muscle thickness of 70 mm^37^, human thigh muscle thickness^38^, ^39^ of ~44 mm from the skin to the nearest prominent bone, and breast tissue of 40-50 mm average thickness during mammographic screening. Such an accessible range could be relevant to *in vivo* human breast cancer, head and neck tumours and prostate cancers. A lateral spatial resolution of approximating 50% of the thickness of the tissue of similar TRS systems reported earlier by our group^23^, ^40^ suggests the ability to discriminate multiple tumours in proximity and a potential indication of the tumour sizes. Detection of SERS NPs through a heterogeneous tissue comprising of protein, lipid, blood, bone, and skin, further strengthens the future scope of SEDRS biomedical applications.

#### 2.3.3 Tumour detection from tissue depth (Z-plane) by inverse SORS as the DRS

**Figure 4:**
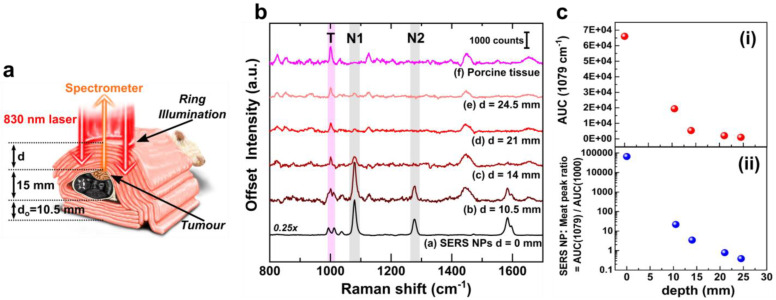
(a) Schematic showing the SORS SEDRS measurements with ring illumination and point collection, the cross-section is shown for better understanding, while all actual measurements were performed on the whole animal. (b) Spectra at various depths d with marked peak position from porcine tissue T and BPT N1 and N2, and (c) area under the curve AUC of peak Y at 1079 cm^-1^ with respect to (i) depth d and (ii) AUC ratio of NP: Tissue log plot.

Similar depth detection studies on the post mortem mouse were carried out using an inverse SORS set-up with a ring illumination and backscattered point collection positioned at the centre of the ring, resulting in a 5 mm spatial offset (maximum available with the existing set-up). In the inverse SORS modality, both the laser photons and Raman photons travelled through the same tissue thickness d to reach the tumour (with the mouse oriented such that the tumour was on top of the mouse body, as opposed to the TRS measurements) (see figure 4a). The thickness d was increased from 10.5 mm to 25 mm with an increment step of 3.5 mm. The lower layer tissue thickness d_o_ (below the mouse) was kept constant at 10.5 mm as used in TRS modality. Like TRS, at each depth, three individual spectra of 10 s accumulation were collected, averaged and then a polynomial baseline was subtracted in OrginPro software. The spectra are plotted in figure 4. Additional raw spectra in figure S5 and spectral post-processing method in figure S6 (in Supporting Information) have been shown for SORS modality, similar to that of TRS. Figure 4b confirms the inverse relation of the SERS NP Raman intensity and the tumour depth d, like that observed for TRS (figure 3). The signal was experimentally detected at a maximum depth of 24.5 mm with an SNR of 2.27. This detection depth was comparable to that achieved by Faulds and co-workers^9^ for resonant SESORS with a higher 8 mm offset (as compared to the non-resonant SESORS detection with 5 mm offset in this study). Such SEDRS SORS system holds promise for *in vivo* human epithelial and breast cancer detection. Further instrumentation modifications are being explored to surpass the achieved depths by optimizing the spatial offset.

Comparing the present SORS and TRS set-ups reveals that the signature peak of porcine tissue T was significantly stronger in inverse SORS than in TRS modality, stemming from the radical difference in experimental geometries. Figure 4c(i) shows the decrease of area under the curve (AUC) of the peak N1 as a non-linear decay with increasing depth, as opposed to the linear decay observed for the TRS modality (figure 4d-iv). With an increase in depth, a ratio of AUC of BPT to that of Phe (N1:T) (figure 4c-ii) confirms that BPT signal's contribution becomes weaker compared to that of the surface layer of porcine tissue. The maximum depth of detection achieved using the inverse SORS modality was significantly less than that in TRS modality. This difference arises due to the modality geometries and the sample arrangements (i.e., tumour position from both laser and Raman side) of a modality^3^. Depth interrogation utilizing transmission and inverse SORS was further compared to conventional backscattering point-modality Raman. This revealed that the sub-surface to surface signal discrimination, although higher for transmission modality, was impacted by the sample orientation and tumour positioning, as explained in section 6 and figure S7 of the Supporting Information. Therefore, each modality demonstrates strengths and weaknesses and should be chosen based on specific end-use.

## 3. Conclusions

In this study, we investigated the detection of cancer tumours in a mouse model using deep Raman spectroscopy and concluded that while a primary SERS NP dose was sufficient for surface tumour detection, a booster NP dose was required for detection through additional layers of mammalian (porcine) tissue. The SERS NP imaging agent (here, BPT-labelled gold NPs) acted as a location identifier of the breast cancer tumour in the mouse, distinguishing it from surrounding soft tissues. The NP concentration-dependent SERS signal intensity allowed for precise tumour location identification spatially (2D) even within a few mm^2^ scan areas. This is critical for ascertaining follow-up treatment efficacy, as well as help in multiplexed cancer sub-type detection. The retrieval of SERS signal from within a tumour accurately from all three dimensions (spatial 2D and depth) makes the technique suitable for progressing cancer tumour diagnosis. To this end, the advances of deep Raman spectroscopy imaging modality when combined with a SERS imaging agent, i.e., SEDRS brings forth greater potential for non-surgical Raman cancer diagnosis. A combined dose (primary and booster) of about 10^10^-10^11^ of BPT-labelled SERS gold NPs allowed identification of their signature BPT peaks from a maximum tissue thickness of 71-76 mm in transmission modality, the highest reported to date. Such thicknesses are comparable to human tumour depths, especially for breast cancers, head and neck cancers, and prostate cancers. A moderately quick 10 s detection through heterogeneous tissue comprising of skin, bone, blood, organs, protein, and lipid while probing *in vivo* relevant NP concentrations demonstrate the potential clinical scope of SEDRS cancer imaging modality. The transmission SEDRS modality was most effective in signal suppression of the surface component and the relative enhancement of the signal contribution from the sub-surface component, for the experimented conditions. Although it should be noted that the compact system utilized only a limited and suboptimal maximum spatial offset of 5 mm, the backscattered inverse SORS modality still permitted the collection of signals from a depth of 24.5 mm. Improvements in the instrumentation and SERS nanostructure are anticipated to increase the detection depth further to make it comparable with ultrasound imaging with the added benefit of high chemical specificity and sensitivity of Raman spectroscopy - an ideal combination for future clinical cancer imaging.

## 4. Methods

***NP functionalization and properties.*** Citrate-capped spherical gold nanoparticles of 60 nm diameter were purchased from nanoComposix. The product specifications: TEM diameter of 60 ± 6 nm, LSPR peak at 532 nm, the mass concentration of gold of 0.053 mg/mL with 2.4×10^10^ NPs/mL. It was functionalized with a Raman label Biphenyl-4-thiol (BPT) and a linear polyethylene glycol (PEG) thiol polymer of 3000 Da molecular weight. A 4:1 molar ratio of BPT (100 µL of 1 mM) to the polymer (25 µL of 1 mM) was used for 1 mL of the as-obtained NP, such as to provide near 100% coverage of the nanoparticle surface. The colloid was centrifuged after 20 minutes, at 4000 rpm for 10 minutes. The supernatant was removed, and the pellet was re-dispersed in 1 mL milliQ water such as to provide the identical NP concentration with respect to that of the supplied. By varying the re-suspended milliQ water volume, the concentration was adjusted. The LSPR shifted to 534 nm and no secondary peak was observed, suggesting that unwanted aggregation had not occurred. The median hydrodynamic diameter was measured using a Malvern DLS Zetasizer and observed to be 90 nm with a FWHM of 70-150 nm (see Supporting Information figure S3). This would also aid in providing a steric stabilization envelope for the NPs and frustrate random aggregation between them, avoiding SERS intensity fluctuation. The absorbance at 450 nm (A_450_) was obtained using a UV-Visible spectrometer and the NP concentration calculated using the relation of concentration c mol/L = A_450_/ε_450,_ where ε_450_ for 60 nm diameter gold NP was considered to be 1.73×10^10^, reported by Haiss *et. al.*^41^ This concentration value was converted to total NPs using the final injection volume of the functionalized gold NPs, i.e., for the 1^st^ injection of volume 200 µL containing NPs with a total of 1.5×10^9^ NPs. The 2^nd^ injection comprised of 100 µL containing a total of 7.2×10^11^ NPs.

### 4.1 Syngeneic tumor model

All animal research was conducted under a UK Home Office license. All protocols were reviewed and approved by a UCL local ethics committee. Six to eight weeks old female BALB/cAnNCrl mouse was purchased from Charles River Laboratories. Animals were housed five per cage in Individually Ventilated Cages and habituated to the animal unit for 7 days before the start of the experiment. The animal was allowed access to standard rodent chow and water *ad libitum* and lighting were controlled on a twelve-hour cycle (on at 07.00 h and out at 19.00 h) at an ambient temperature of 22 °C (22-24 °C) and humidity of 45%. Animals were randomized before all experiments by weight (weight approximately 19-23 g) and acclimatized to the procedure room for 1 h before performing scientific procedures. 4T1 tumours were induced as described in earlier work^42^. Briefly, one million 4T1 mammary carcinoma cells (purchased from American Type Culture Collection) at passages 3-10 in 100 μL of sterile RPMI 1640 medium without Fetal Bovine Serum or antibiotics (Thermo Fisher) were inoculated into the right flank, subcutaneously using a 27-gauge needle. Tumour growth was homogeneous (average 14-day tumour size, 6-8 mm^3^ as measured by digital caliper in both length and width (approximate volume 100-300 mm^3^, average weight 0.3-0.4 g).

***SERS NP injections*. (i) Primary dose as *in vivo* nanoparticle injection.** A mouse was injected subcutaneously distal to the tumour with 200 µL prepared gold NP colloid (approximately, 1.5×10^9^ NPs as calculated from the UV-Visible-NIR spectroscopy), 2 weeks post-induction. Four hours after injection, one mouse was terminated and stored at -80ºC. **(ii) Booster dose as post mortem nanoparticle injection.** The euthanized mouse stored at -80 ºC was thawed. The second injection consisted of the same NPs in a concentrated form (i.e., 100 µL containing a total of 7.2×10^11^ NPs) which was injected subcutaneously (into the same mouse used earlier). This would translate into approximately 7.22×10^11^ NPs as a combined first and second injection.

***Whole animal SEDRS experiments.*** The animal used in these experiments weighed 21.3 g before injection while the dissected tumour weighed 0.31 g approximately 1.45% of body weight (human breast tumours 2-5 cm in diameter weigh 4.2 - 15.6 g, 0.007 - 0.026% body weight). The whole animal was frozen and stored at -80 ºC. Before Raman experiments, the carcass was thawed for 2 hours at room temperature.

### 4.2 Store-bought porcine tissue used for increasing detection depth

Store-bought pork medallions were high in protein composition and low in lipid composition, each of thickness about 3.5 mm. These were used to wrap around the mouse, building up the layer-by-layer thickness of the tissue for detection. The tissue for depth detection consisted of the pork protein tissue, the whole mouse consisting of skin, internal organs, tissues comprised of protein, fat, blood, and bones, resulting in a highly heterogeneous tissue model. The whole tissue was then wrapped with a single layer of cling film and placed on the sample stage onto an optically transparent plate.

### 4.3 Gold distribution in the whole animal by ICP-MS after the second dose

** (i) Sample preparation for elemental analysis by ICP-MS.** After Raman experiments, the whole animal was frozen and shipped to UCL (London, UK) for ICP-MS analysis. The carcass was thawed at room temperature and tumours were excised. Organs: heart, lungs, liver, spleen, pancreas, kidneys, brain and body sections: upper half dissected through the middle along the sternum - termed upper half one and upper half two, lower half - dissected along the midline lower half one and lower half two, contents of the abdominal cavity- termed abdomen, legs- termed leg one and leg two and finally the tail were dissected, weighed (if > 1.5 g they were halved) and minced by scalpel. The tumour was also divided into two parts analyzed separately to check for homogeneity of nanoparticle distribution after the second injection. Then, they were subjected to concentrated nitric acid (70%, 4 mL) and H_2_O_2_ 50% (500 µL) digestion in borosilicate glass tubes (leached with 1% nitric acid at 60 ºC overnight, thoroughly rinsed with Millipore grade water and dried in the air) overnight at room temperature prior heating up to 80 ºC on a heating block for 2 hours. Then, HCl 37% (1 mL) was added, and samples were kept on a hot block for another hour. Digests were diluted 50 times v/v with 5% HCl. Samples were filtered through 0.22 µm Teflon membranes if any particulates were observed.

** (ii) ICP-MS analysis.** Gold concentrations were then determined at the Natural History Museum (London, UK) using an Agilent 7700× inductively coupled plasma mass spectrometer (ICP-MS) to monitor ^197^Au. ^103^Rh was used online as the internal standard. Two calibration curves were prepared as follows: first with 0.5, 1, 5, 10 and 50 ppb (R2 = 0.9998, LOQ = 0.87 ppb) levels and a second with 0.1, 0.5, 1, 5 and 10 ppb (R2 = 1, LOQ = 0.006 ppb) in [He] mode. Samples that were below LOQ in the first run were re-run using the second calibration. It was found that running calibration across the range is impacted by Au's 'memory effect', which accumulates throughout the run and washes into the lower concentration samples. A 10 ppb and 1 ppb standard solution (AAAU1, Inorganic Ventures, US) respectively were run every 5 samples to correct for drift. ^181^Ta was run to check for interference from ^181^Ta^16^O+. The accuracy and reproducibility of the analyses were checked using a calibration check solution (CCS-2, Inorganic Ventures, certified, traceable to NIST) at 10 ppb and 1 ppb respectively.

ICP-MS was run in both no gas and helium (He) mode (5 mL/min He, 99.9995 % purity) to minimize molecular interferences from plasma and solution components. The limit of quantification (LOQ) of the ICP-MS analysis was determined as the concentration corresponding to 10 times of the standard deviation of the signal obtained from the analysis of 5% HCl solutions (seven times) in each run. Gold concentrations (ug/g) are an average of 5 replicate analyses with RSD <5%. In the first run calibrated between 0.5 and 50 ppb, only tumour samples, leg one and upper half two, were above the LOQ of 0.87 ppb. In the second run calibrated between 0.1 and 10 ppb, LOQ = 0.006 ppb, all samples were found above LOQ however, accuracy for this run was poor: CCS-2 at true value 0.94 ppb was measured as 0.13 ppb average throughout the run which is 85.69% different to the true value (this is a consequence to adsorption effects to the plastic (unpublished data). Note, the LOQ and CCS-2 check solution, run every 5 samples, was measured for error and accuracy throughout the run (e.g., CCS-2 true value 9.97 ppb measured 9.54 difference to a true value approximately 5%).

### 4.4 Instrumentation

#### SEDRS Instrument set-up

An in-house built compact DRS system employing an 830 nm laser excitation and the flexibility of switching between three modalities: (i) backscattered conventional point, (ii) inverse SORS with a ring illumination and point collection and (iii) transmission (TRS) with point illumination and collection.

#### Excitation path

The 830 nm laser (Innovative Photonic Solutions) output was coupled through 105 µm core, low OH, stepped index, fibre patch cord (Thorlabs), through a Thorlabs fibre collimator and laser bandpass filters and then:

Either, directly onto the 45º mirror (Thorlabs) and then an 830 nm dichroic filter (Semrock) onto the sample- thus illuminating with a small, collimated beam (approximately 2 mm at sample) for both point and transmission modalities.

Or, collimated onto the centre of a 12.5 mm diameter custom 5º axicon with NIR AR coatings (Thorlabs) and then onto the 45º mirror (Thorlabs) and then an 830 nm dichroic filter onto the sample. This resulted in an illumination ring of approximately 10 mm diameter (Δs=5 mm) and ring thickness of 1 mm (inverse SORS, geometry configuration (ii)).

The laser intensity at the sample surface for this medical diagnostics application was about 2.35 W/cm^2^ for SORS modality with ring illumination and 11.1 W/cm^2^ for both point and TRS modality with 1-10 s exposures on the sample top surface.

#### Collection optical path

a) **Conventional point collection and inverse SORS modalities (i) and (ii):** For both configurations, the collection was over a large spot (around 4.4 mm diameter) from the centre of the ring. The scattered light passed through the dichroic and was collected with an (f=40 mm) lens collimating the light onto a pair of 830 nm edge filters (SEMROCK Razor edge) and then launched with NA matched lens (f=20 mm) onto Ceramoptec bundle of 200 µm core fibres, which had a 2.2 mm fibre bundle circular array to a linear array at the spectrometer entrance.

b) **TRS modality (iii):** The scattered light was collected from the sample's other side from approximately a 2.2 mm spot. This was facilitated with an (f=20 mm) focal length 12.5 mm diameter lens collimating the light onto a pair of 830 nm edge filters (SEMROCK Razor edge) and then launched with an (f=20 mm) NA matched lens onto the Ceramoptec bundle of 200 µm core fibres, which had a 2.2 mm fibre bundle circular array to a linear array at the spectrometer entrance.

Note the etendue of the collection paths in both SORS and TRS are identical, i.e., the solid angle of collection balances the collection area.

#### Detection system

The Kaiser spectrometer (Holospec 1.8i) included a Kaiser 830 nm notch filter, a 100 µm slit, a transmissive holographic grating, and an Andor iDus 420 deep depletion CCD. Calibration was performed with Raman spectra measured from acetylsalicylic acid powder (from Sigma Aldrich) in a quartz cuvette acting as a calibration standard.

#### Raman spectrum acquisition and analysis

All samples were placed on an optically transparent plate for measurements. The porcine tissue reference spectrum was measured by using two layers of the tissue i.e., 6 mm thickness with point modality (as shown in figure S5 in Supporting Information). Various sample positions for collection of reference, surface and deep Raman signals have been pictorially shown in figure S4 in Supporting Information. The spectrum was acquired using Andor Solis software and analyzed using OriginPro software.

Unless otherwise mentioned, most point-modality measurements were obtained as 1 accumulation of 1 s and SORS and TRS at 10 s acquisition times. Each sample position (for point, SORS and TRS) was measured thrice (i.e., 3 individual spectra with minimal sample movement between these measurements) and an averaged spectrum from these (equivalent to 1 or 10 s accumulation, respectively) was used for further analysis for all surface position and depths studied. A polynomial estimate of the background, simulated in OriginPro, was then subtracted from this, providing a background-subtracted spectrum for each depth (as elaborated in figure S4 and S5 in Supporting Information). This averaged, background subtracted, unsmoothed spectrum was then studied for each depth d. Furthermore, the area under the curve (AUC) for peak N1 at 1079 cm^-1^ was calculated by summing the intensity over the simulated background and plotted with respect to depth.

## Supplementary Material

Supplementary figures and tables.
