# Supplementary material for: Surface enhanced deep Raman detection of cancer tumour through 71 mm of heterogeneous tissue
Source: Nanotheranostics. 2022 Mar 21;6(3):337–49. doi: 10.7150/ntno.71510 (PMC9194587; doi:10.7150/ntno.71510)
Supplement: Supplementary file 1 — Supplementary figures and tables. [file ntnov06p0337s1.pdf]

Supporting Information

for

Surface enhanced deep Raman detection of cancer tumor through 71 mm of heterogeneous tissue

Priyanka Dey[1,2], Alexandra Vaideanu[3], Sara Mosca[4], Marzieh Salimi[1], Benjamin Gardner[1], Francesca Palombo[1], Ijeoma Uchegbu[3], Jeremy Baumberg[5], Andreas Schatzlein[3], Pavel Matousek[4,*] and Nick Stone[1,*]

Affiliations

[1]School of Physics and Astronomy, University of Exeter, Exeter, UK
[2]School of Health and Life Sciences, Teesside University, Middlesbrough TS1 3BX, UK
[3]School of Pharmacy, University College London, London, UK
[4]Central Laser Facility, Research Complex at Harwell, STFC Rutherford Appleton Laboratory, UK Research and Innovation, Harwell Campus OX11 0QX, UK
[5]NanoPhotonics Centre, Cavendish Laboratory, Cambridge, CB3 0HE, UK

*Corresponding authors: PM: pavel.matousek@stfc.ac.uk and NS: n.stone@exeter.ac.uk

<u>Contents:</u>

1. Deep Raman spectroscopy (DRS) instrumentation set-up in various modality
2. SERS nanoparticle imaging agent
3. ICP-MS data of the mouse after the combined injection of primary and booster dose
4. Sample positioning during SEDRS measurements
5. Raw SEDRS spectrum and spectral post-processing
6. Analytical approach towards DRS modality selection

1. Deep Raman spectroscopy (DRS) instrumentation set-up in various modalities

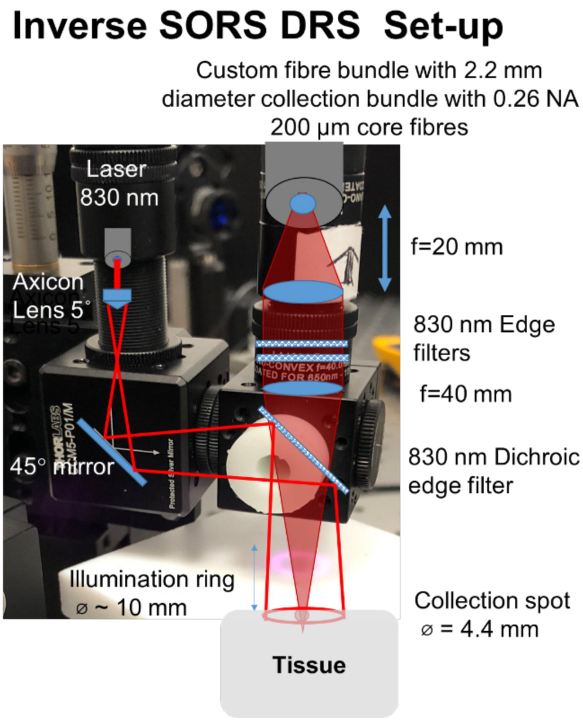

Figure S1. Deep Raman Inverse SORS modality

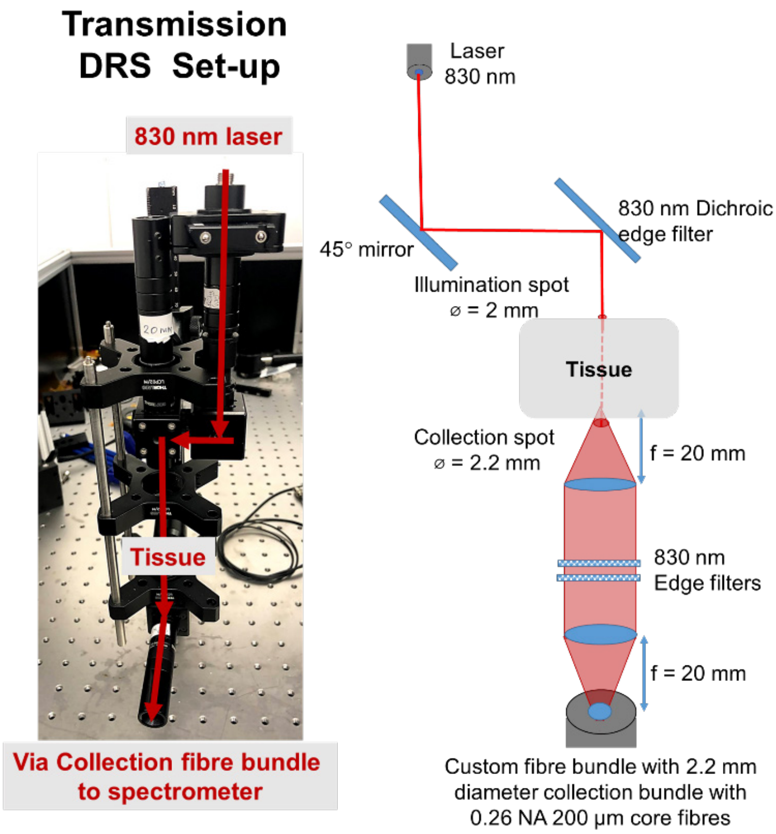

Figure S2. Deep Raman transmission TRS modality

2. SERS nanoparticle imaging agent

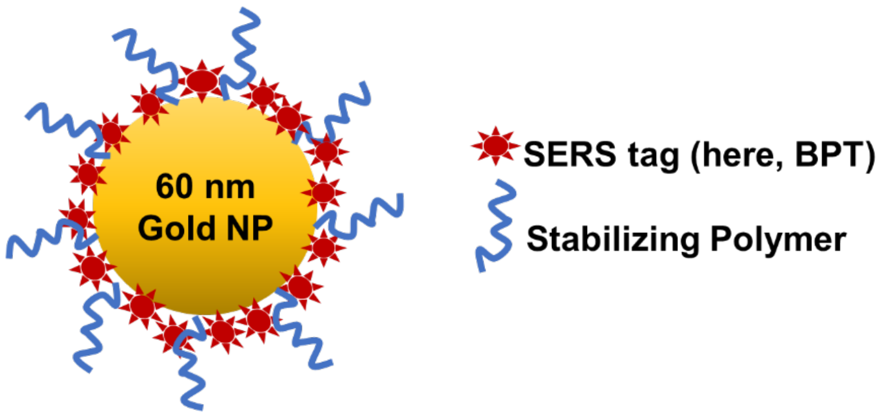

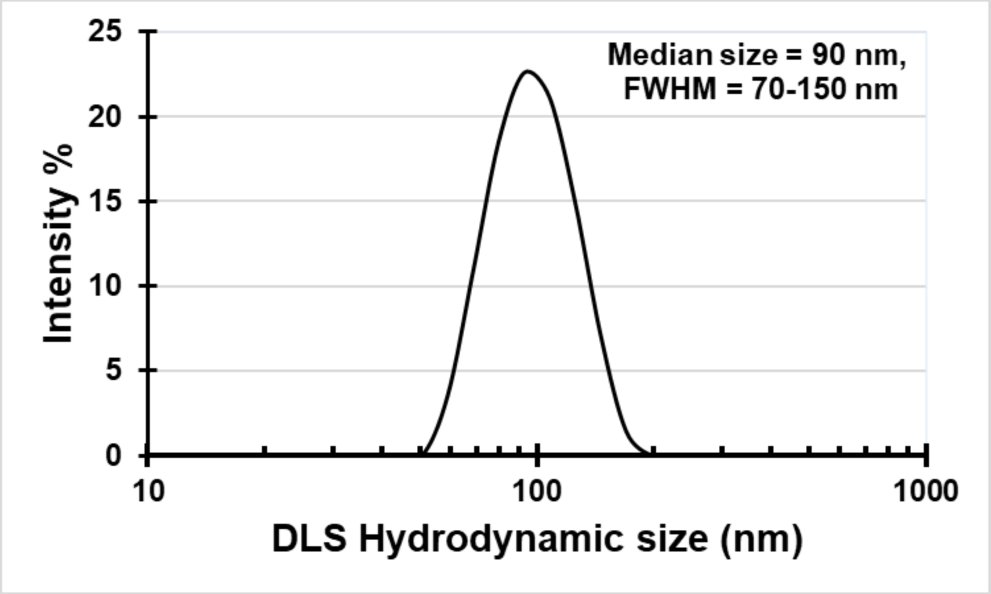

Figure S3. Cartoon representation of the SERS NP employed for this study. Dynamic Light Scattering (DLS) plot depicting the diameter of the same in the colloid.

3. ICP-MS data of the mouse after the combined injection of primary and booster dose

Table S1. Tabulated data of ICP-MS measurements organ and sample-wise.

Organ name	Organ ID	Sample description	Measured conc (ppb)	Volume digest (mL)	Au mass (µg)	Au % total measured	Au % total measured (plotted)
Heart	S1	Heart	8.40	5.076	0.043	0.17	0.17
Lung	S2	Lung	7.55	5.165	0.039	0.15	0.15
Kidney	S3	Kidney	7.70	5.135	0.04	0.16	0.16
Liver	S4	Liver_1	8.60	5.200	0.045	0.18	0.37
	S5	Liver_2	9.20	5.347	0.049	0.19	
Tumor	T1	Tumor_1	2312.68	5.041	11.658	45.42	63.86
	T2	Tumor_2	942.97	5.020	4.734	18.44	
Pancreas	S8	Pancreas	6.80	5.091	0.035	0.14	0.14
Spleen	S9	Spleen	8.40	5.132	0.043	0.17	0.17
Brain	S10	Brain	8.85	5.125	0.045	0.18	0.18
Tail	S11	Tail	8.35	5.310	0.044	0.17	0.17
Head	S12	Head_1	17.50	5.920	0.104	0.41	0.22
	S13	Head_2	9.15	6.158	0.056	0.22	
leg_ tumour-bearing	S14	leg_tumour-bearing	1114.47	5.731	6.387	24.88	24.88
leg_ contralateral	S15	leg_contralateral	9.30	5.755	0.054	0.21	0.21
abdomen	S16	abdomen_1	9.60	6.238	0.06	0.23	0.46
	S17	abdomen_2	9.60	6.108	0.059	0.23	
upper	S18	upper_1	26.05	6.622	0.173	0.67	7.50
	S19	upper _2	266.95	6.562	1.752	6.83	
lower	S20	lower_1	18.65	6.465	0.121	0.47	0.97
	S21	lower_2	10.70	6.438	0.069	0.27	
	S22	lower_3	9.80	5.872	0.058	0.23	
		**total measured**			**25.668**		**99.59**

4. Sample positioning during SEDRS measurements

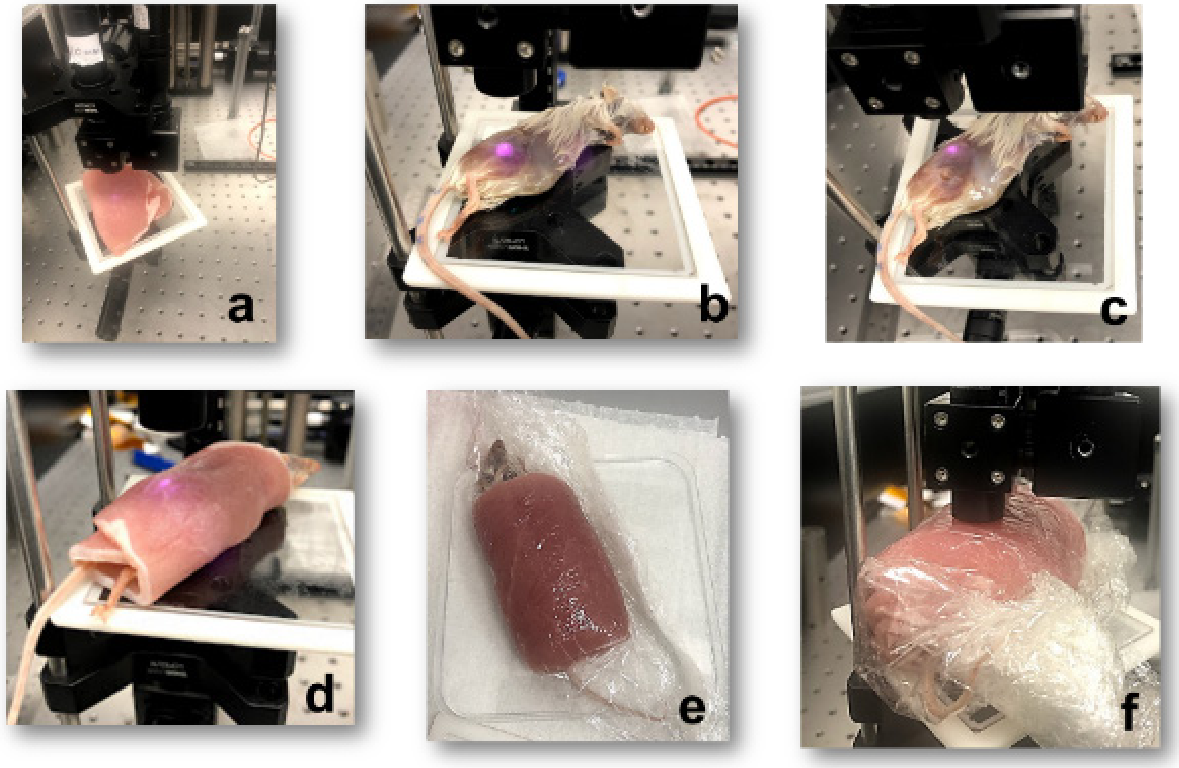

Figure S4. Photos to show the sample set-up in different scenarios supported on a near infrared transparent glass holder: (a) only porcine tissue for reference spectrum, (b) mouse conventional Raman point modality on tumour, (c) mouse conventional Raman point modality away from tumour, (d) mouse wrapped with porcine tissue layers with conventional Raman point modality, (e) mouse wrapped with multiple porcine tissue layers and held together in place with a cling film wrap, (f) measurement of sample e in SORS modality.

5. Raw SEDRS spectrum and spectral post-processing

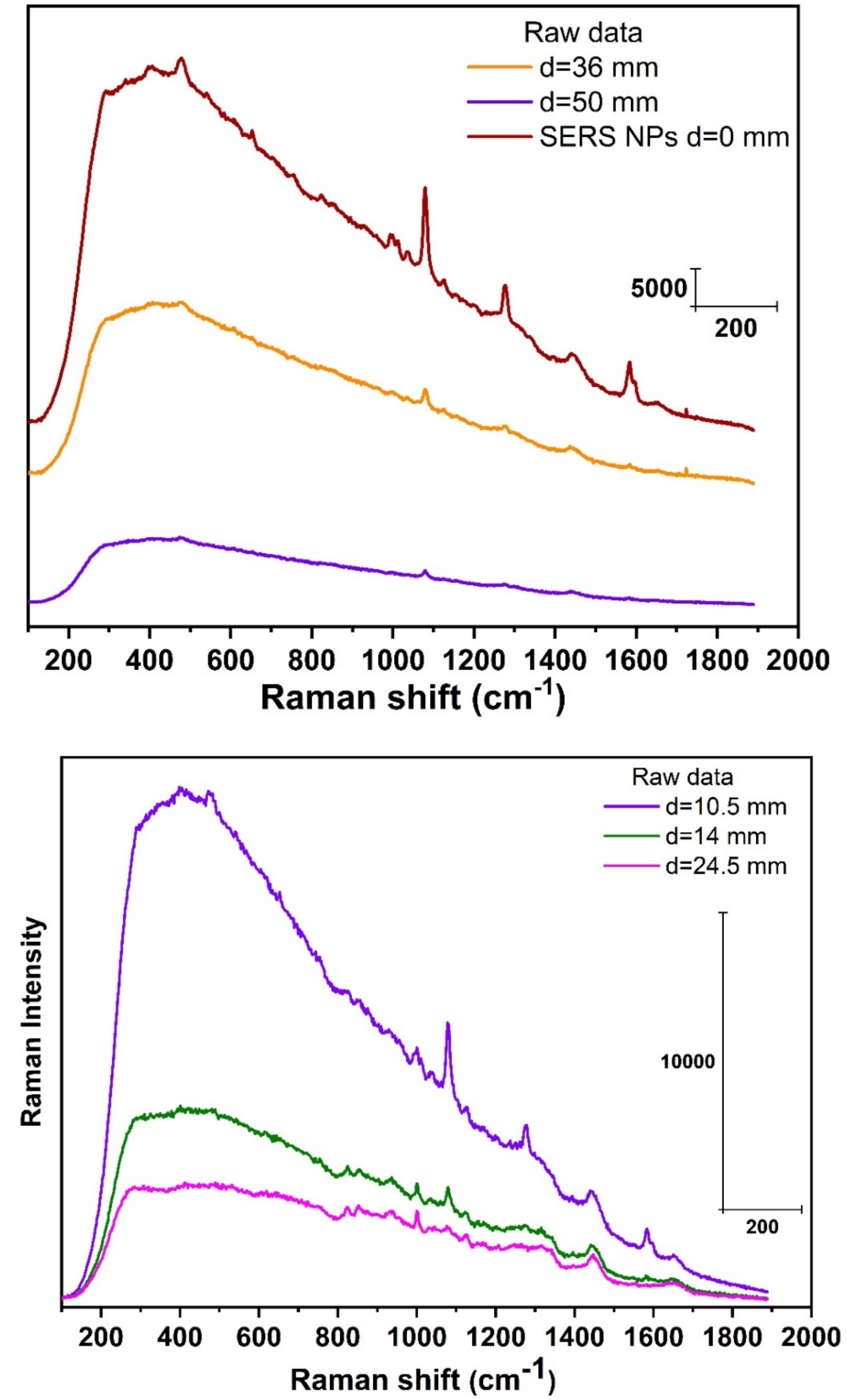

Figure S5. Raw spectrum at different depths. ***(Top)*** TRS modality and ***(Bottom)*** SORS modality.

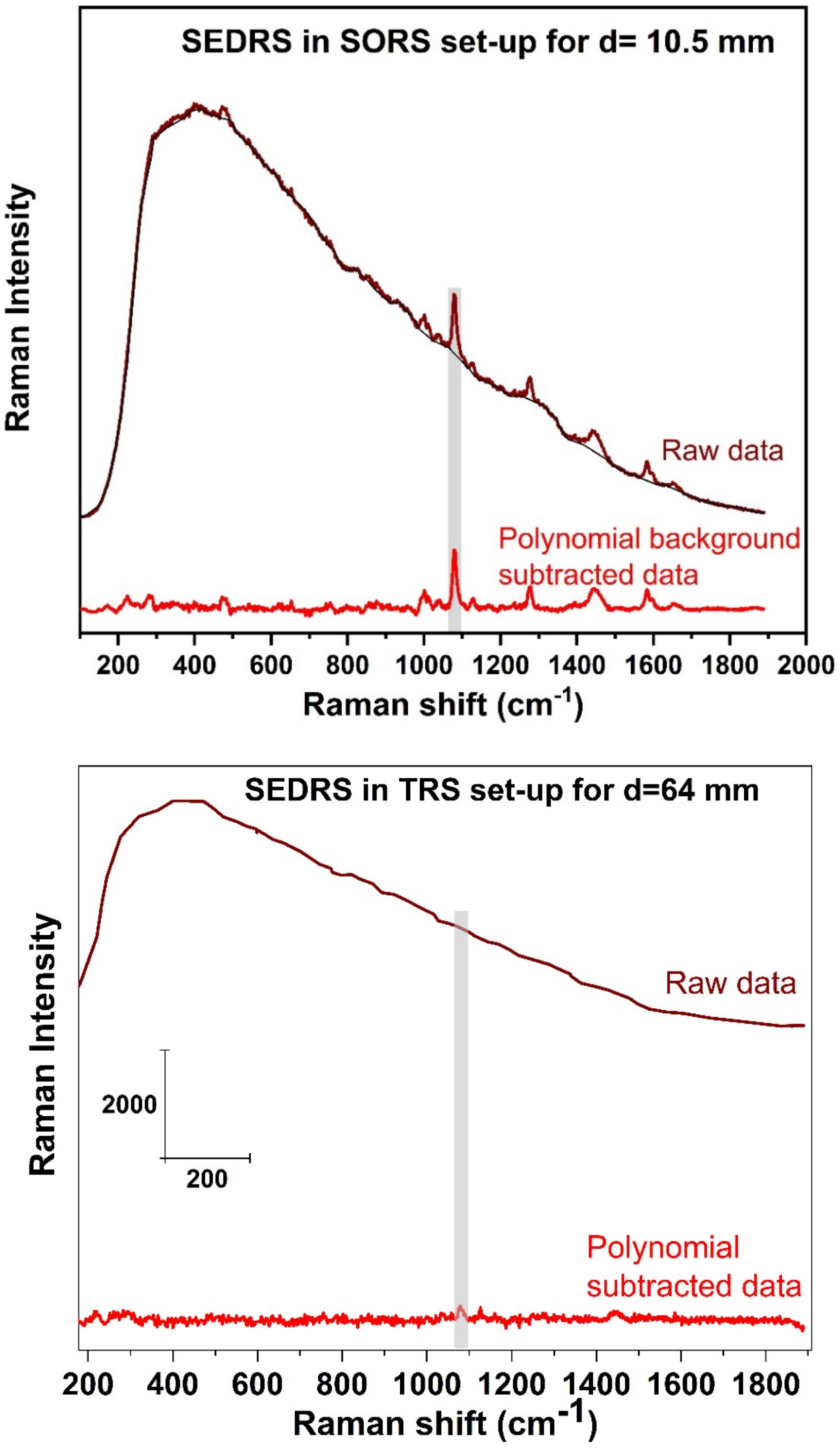

Figure S6. Spectral post-processing. Polynomial background subtraction from averaged spectrum (average of 3 spectra at same depth) in OriginPro resulting in the final spectrum at each depth, which has been used for analysis and shown in the main text. No smoothing done. ***(Top)*** SORS modality with black curve demonstrating the simulated polynomial background, and ***(Bottom)*** TRS modality. The grey shaded bar shows the position of the signature BPT peak at 1079 cm^{-1} (N1).

6. Analytical approach towards DRS modality selection.

The differentiation of the surface and subsurface composition that the three modalities probed demanded further investigation. So, we compared the spectrum and the AUC of the relevant signature peaks of NP to that of tissue (N1:T). Each of the modalities features a different optimized sample arrangement which allows the highest signal retrieval, which makes direct comparison challenging. Therefore, the spectrum for each modality was selected such that the total tissue thickness (conventional point-modality and SORS: 39.5 mm and TRS: 36 mm) and depth of tumour from the nearest tissue surface was similar (conventional point-modality and SORS: 14 mm and TRS: 10.5 mm on Raman side). Figure 5 compares the background-subtracted spectrum in the SORS modality (orange spectrum), conventional Raman point-modality (green spectrum) and TRS modality (blue spectrum). The ratio of the AUC of the BPT SERS NPs to that of the tissue (i.e. N1:T) was approximately 0.5 for point-modality, increasing to ~1 for SORS, confirming that the small offset SORS set-up probes sub-surface components while still detecting significant surface components. An interesting feature to note is that the tissue peak T at 1000 cm^{-1} was significantly subdued in TRS, while present dominantly for both point and SORS modalities. The signature peak of BPT at 1079 cm^{-1} (N1) was present for all modalities, providing an AUC ratio of N1:T of more than 10 for TRS modality. Thus, for the studied parameters, the TRS modality allowed higher discrimination of sub-surface signal to that of surface signal than the SORS modality, with the least discrimination obtained from the conventional point-modality. It should be stressed that for backscattered SORS and conventional Raman, the distance from the top surface to the tumour is crucial, whereas for TRS the overall sample thickness is more critical. For example, if the tumour was close to the surface, then SORS and conventional modalities would detect it and the remaining sample thickness would not be very relevant. In comparison, for TRS the overall sample thickness, if too high, could easily preclude such a simple measurement. This confirms that although the TRS probed higher detection depths, the choice of a SEDRS modality should be done based on the specific end-use and sample arrangement i.e., tumour accessibility.

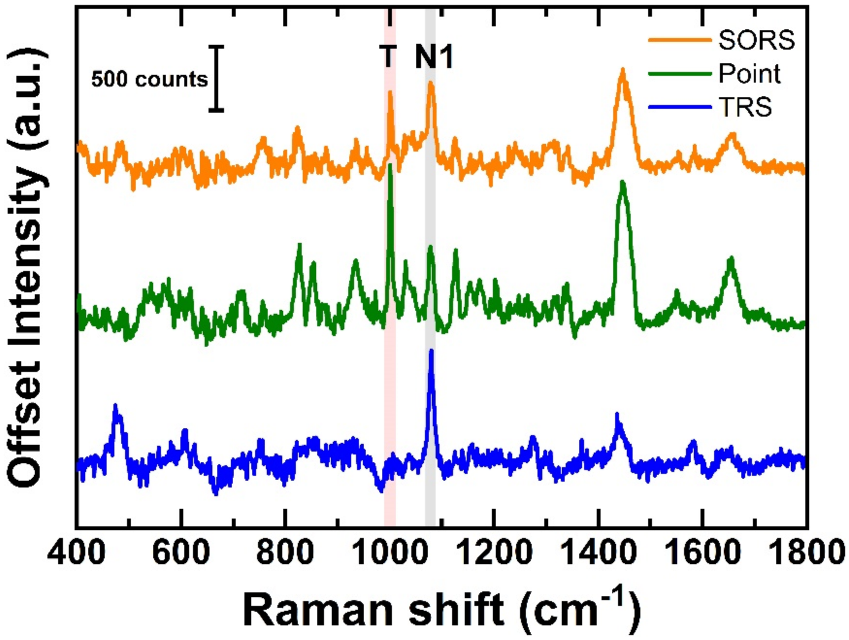

Figure S7. Comparison of different modalities. SORS (orange spectrum) and point modality (green spectrum): depth of tumour = 14 mm, total thickness = 39.5 mm, whereas, for TRS mode (blue spectrum): depth of tumour from nearest tissue surface on Raman side = 10.5 mm, total thickness = 36 mm**.**
